# Evolving Cityscape: A Dataset for Building Footprints and Heights from Satellite Imagery in China

**DOI:** 10.1038/s41597-025-05971-0

**Published:** 2025-10-23

**Authors:** Sebastiano Papini, Susie Xi Rao, Peter H. Egger

**Affiliations:** 1 Chair of Applied Economics, ETH Zurich, Leonhardstrasse 21, Zurich, 8092 Switzerland; 2 ETH AI Center, Zurich, Switzerland; 3 CEPR, London, UK; 4 CESifo, Munich, Germany; 5 Leverhulme Centre for Research on Globalisation and Economic Policy (GEP) at the University of Nottingham, Nottingham, UK

**Keywords:** Economics, Geography, Interdisciplinary studies

## Abstract

The study of cities faces a core challenge: the absence of data that are simultaneously high-resolution, large-scale, and longitudinal. Only combining these three aspects reveals detailed (almost building-level) changes while covering vast urban areas consistently over time and promises advancing our understanding of the driving mechanisms of spatial agglomeration. We present a novel approach that leverages computer-vision techniques on Sentinel satellite imagery to generate detailed building-volume data throughout 106 cities in China over a six-year period (2018-2023). We validate the model by assessing building-volume density in out-of-sample cities. Additionally, we compare our results to nightlight-luminosity data, a frequently utilized remote-sensing resource for tracking density and human activity, and demonstrate how the proposed method and data drastically improve the measurement of urban density. The proposed method provides researchers in the social sciences at large with access to large and exponentially growing archives of customary daylight-satellite imagery either through direct use of the provided dataset or through adaptation of the model with new data.

## Background & Summary

A key challenge in the social sciences dealing with meso- and micro-regional development is the complete or at least vast lack of large cross-sectional and sufficient longitudinal coverage of high-resolution data on the make-up of micro-regions, in particular, in cities and metropolitan areas. One would wish to use such data to study the extent, location, and, eventually, the determinants and consequences of human spatial configurations and the artificial environment they constitute.

However, if available at all, such data mainly exist for regions where the development process has already matured, namely in industrialized countries such as the United States^1,2^, Germany^3^, or England^4^. In the light of a recent interest in cities in developing countries^5–7^, demand arises for longitudinal data of sufficiently many regions to learn systematic patterns about the urbanisation in contexts with a rapid urban-industrial transformation. After all, average causal relationships can more likely be established, where sufficiently big changes occur in the data, and where the cross-sectional and longitudinal support is sufficient in permitting some generalization. Additionally, there is a broader global reorientation of economic activity and human settlement patterns, with the center of gravity increasingly shifting toward the rapidly transforming regions of the Global South^8^. This paper aims at providing a pipeline to generate data on the micro-structure of cities by utilizing modern methods in computer vision in conjunction with satellite imagery. Specifically, the data we provide are of medium-high resolution on a  ~10 × 10 m grid and, hence, fine-grained enough to map building structures at the pixel level, covering 106 cities in China annually between 2018 and 2023.

China is an economy that fits the purpose very well. Its economy grew at a much faster rate than data-rich industrialized countries since its opening up to foreign trade in 1978 and, in particular, since the early 1990s. That era constituted a time of still ongoing substantial change from an agricultural non-market economy to one of the leading manufacturers in the global market and with its fundamental transformation in the distribution of its population between rural areas and agglomerations (cities and metropolitan areas). What is unusual is that all of that – including the urban transformation – happened in a time span when high-quality remote-sensing data were available. However, China’s rapid transformation has also brought about challenges related to pseudo-urbanization, where urban expansion and infrastructure growth sometimes outpace the actual integration of rural migrants into the urban economic and social fabric^9^.

One particular line of interest in urban change and agglomeration is devoted to the question of scale. In particular, one line of research aims at understanding patterns of multi-scalability^10^ – here, how urban processes manifest across different spatial levels. Related work emphasizes the importance of scale-sensitive analysis for understanding socio-spatial systems. Obviously, the pursuit of such a question requires high-resolution, longitudinal data to be able to distinguish patterns at a wide range of spatial aggregates, from micro to macro. Methodologies like the one presented in this paper support the study of multi-scale dynamics of urbanization, inequality, and spatial structure, especially, in contexts of rapid transformation.

Daylight satellite imagery used in conjunction with deep-learing methodologies enables a standardized, fine-grained measurement of urban density and functional growth across space and time. To this end, LiDAR offers a high-quality three-dimensional mapping^11^. But it is very costly to be used on a large scale and with repeated time observations per spatial unit^12,13^. Traditional machine learning methods can help estimating building heights from widely (and cheaply) available optical and radar images^14–16^, but they typically need to rely on expert-driven feature engineering, which limits the transferability of their pipeline to other settings. Deep learning offers a more scalable and transferable alternative, which learns patterns directly from imagery and captures spatial patterns through convolutions. It also enables retrospective analysis of urban patterns using historical satellite archives. Yet, despite its potential, time-series estimates of high-resolution urban patterns at the building level are scarce^17–19^, largely due to the lack of longitudinal reference data.

In recent years, a new strand of interest and research in remote sensing has emerged, focusing not only on the measurement of building footprint and land-use intensity but also that of building height (or volume). An inspection of this body of literature suggests the following trade-off: data with a fine granularity that permit the measurement of building footprint and height at the level of individual buildings^16,20–25^ are typically only available cross-sectionally and often with limited accessibility for research; data with longitudinal coverage^15,26–28^ are available either for coarse levels of granularity or at severely limited cross-section. Hence, existing data do not support research on the three-dimensional (3D; building height and volume) micro structure of large inter-city cross sections with substantial changes in urban density.

The present paper establishes a deep-learning pipeline to collectively learn building footprint and height at sufficiently fine granularity, permitting a cross-sectionally broad coverage and a longitudinal dimension. We employ this pipeline and publish the resulting extensive data set that covers 106 Chinese cities over 6 years (2018-2023). We first collect daylight satellite data of Sentinel with a resolution of  ~10 × 10 m and the corresponding 3D reference data in 40 cities. We then use a multi-task learning approach to predict the building footprint and height in the same framework: (1) we determine the surface area covered by any buildings (the square footage of occupied land); (2) and we determine the height (and volume) of buildings from the imagery. Note that building height on a fixed grid is a linear transformation of building volume. Therefore, we will use the two terms interchangeably at times in this study. We evaluate the popular U-Net architecture with various loss functions, transformer models, and training configurations to predict the two outcomes interest, building footprint and height/volume, on both the pixel and more aggregated levels. We also assess the quality of the building-volume prediction in Shenzhen as a case study. Moreover, we compare our prediction with those generated by existing models targeting building footprint and height. Finally, we assess the difference between our prediction and one obtained based on night-light data to demonstrate the improvement in measuring urban density over a measure used in several disciplines such as economics.

## Methods

**Fig. 1:**
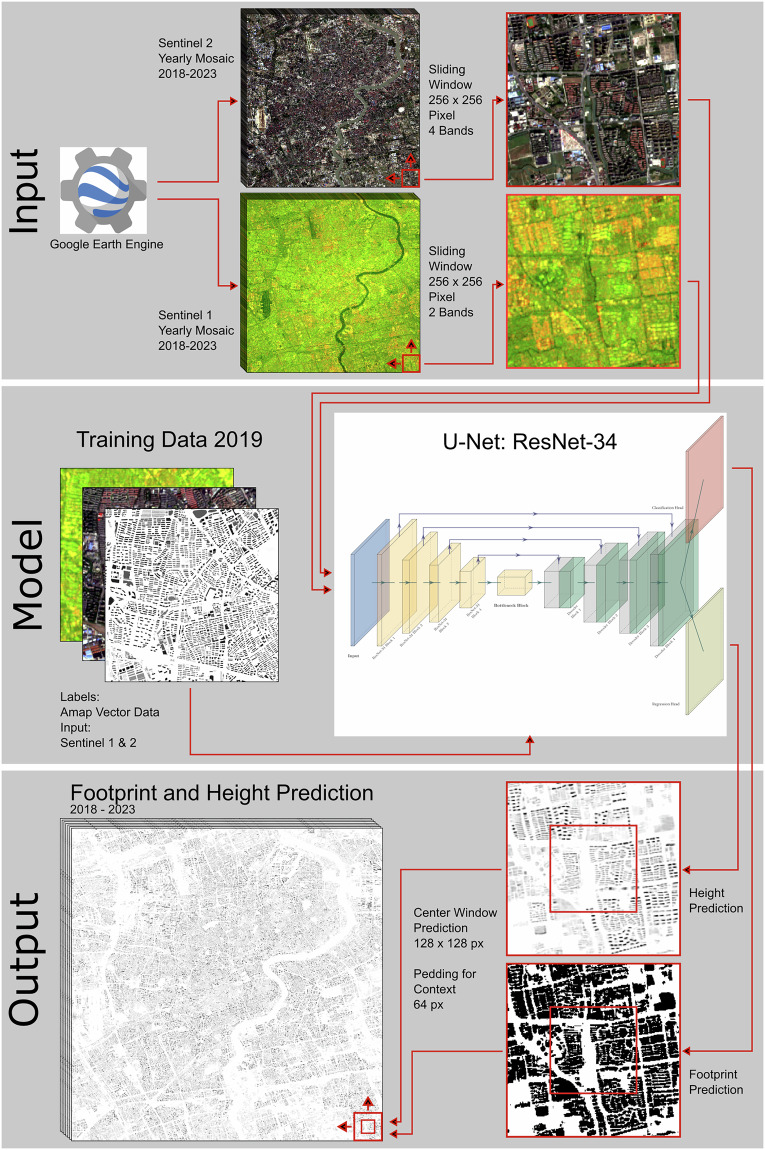
Flowchart of the Prediction Pipeline.

The entire pipeline is visualised in Fig. 1, including model input, training data preparation, multi-task learning, output normalization, and data augmentation.

### Input data

We use Sentinel satellite imagery as our main data source and benefit from its open-access and free-of-charge policy. The image sets include both Sentinel-1 (synthetic-aperture radar, SAR) and Sentinel-2 (multi-spectral optical) products which both provide almost global coverage at a medium-high spatial resolution. We download the data and implement image processing through the Google Earth Engine API.

For Sentinel-2 optical images, we select the level 2A product which provides orthoimage Bottom-Of-Atmosphere (BOA) corrected surface reflectance data. The bands we use contain B2 (blue), B3 (green), B4 (red), and B8 (NIR), with a spatial resolution of  ~10 × 10 m. To reduce the impacts of cloud occlusion, we first filter out images with more than 60% cloud coverage and further apply the cloud mask by making use of the additional Sentinel-2 cloud probability image collections^29^. We query the data in December for Sentinel-1 and annually for Sentinel-2 and use the mean values from multiple returned images to obtain a single image mosaic, covering the area of interest for each city.

For Sentinel-1 radar images, we select the available VV and VH polarizations acquired from a C-band SAR sensor with combined ascending and descending orbit directions to collect maximum information content. For each year, the same image download and pre-processing technique as for Sentinel-2 is applied.

### Training data

As labels we use a reference data set^30^ that is collected by field investigation and published by a popular Chinese online map platform and widely used in the remote-sensing community^20^. It provides vector-data polygons of individual buildings with assigned floor counts. We manually evaluated the quality of the reference data. There are substantial differences in data quality across and within cities. For training of the algorithm, we focus on 37 Chinese cities with the best reference-data quality. These cities were selected based on quality according to the evaluations carried out by^20^ and a sufficient good quality of footprint in cities via visual inspection of the mapping between reference data and satellite images of individual buildings. These cities in the training sample are Baoding, Cangzhou, Chengdu, Foshan, Fuzhou, Guiyang, Hohhot, Hongkong, Huizhou, Jiaxing, Langfang, Linyi, Nanchang, Ningbo, Qinhuangdao, Quanzhou, Sanya, Shengyang, Suzhou, Taizhou, Taiyuan, Tangshan, Weifang, Weihai, Wenzhou, Wuhu, Wuxi, Xi’an, Xiamen, Xuzhou, Yangzhou, Yantai, Yinchuan, Zaozhuang, Zhenjiang, Zhongshan, and Zhuhai. The training data pertain to the year 2019. The cities in the training sample are a subset of the 106 Chinese cities of interest in this study.

**Fig. 2:**
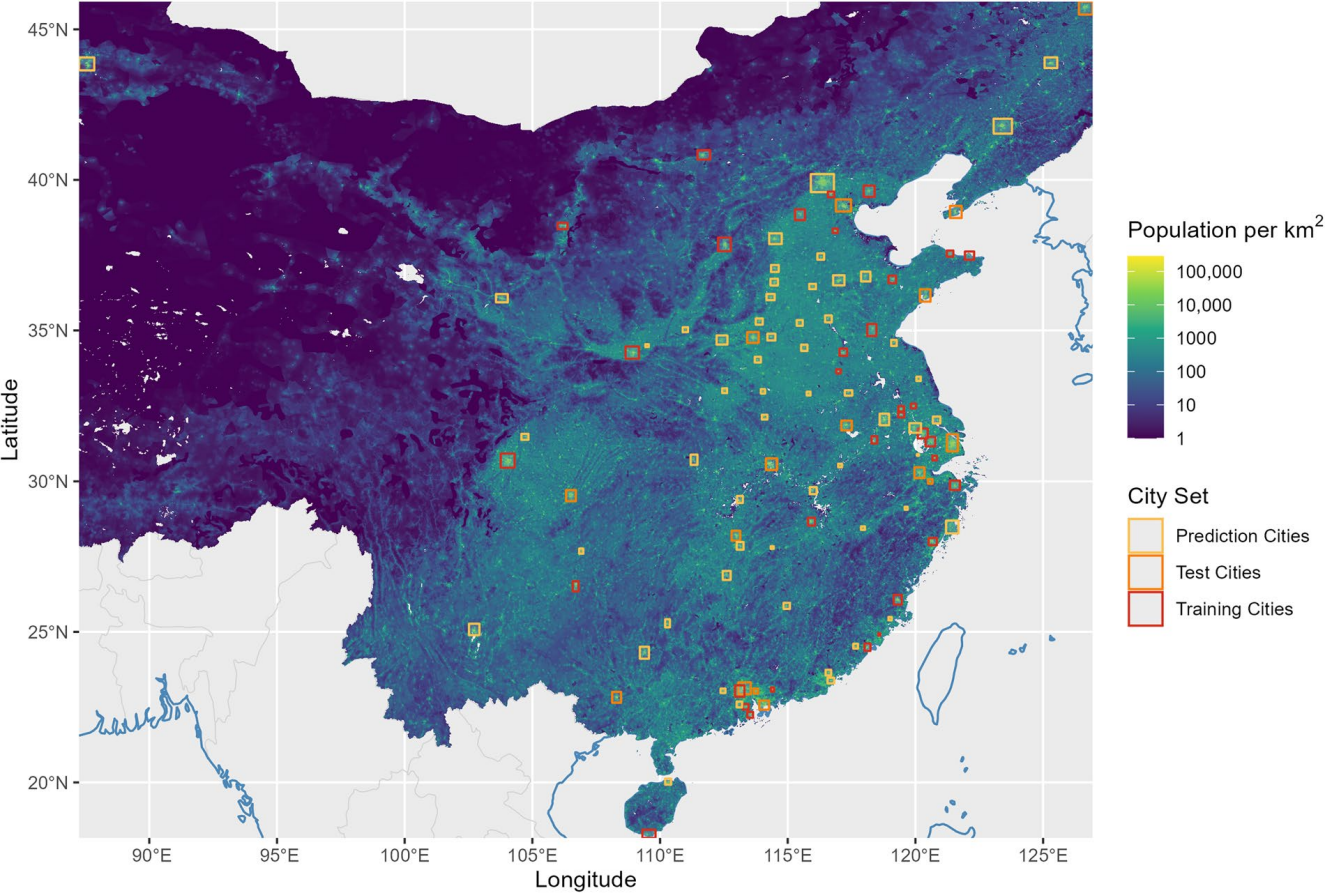
Map of 106 City Bounding Boxes in Mainland China in our Data Set. Note: Coastlines in blue and country borders in gray are drawn using Natural Earth. Population density is based on WorldPop Mainland China estimate^54^.

To prepare training data, the reference data are rasterized from building-level polygons to the same  ~10 × 10 m grid that the Sentinel imagery predetermines. We partition these images by a grid with a cell size of 256 × 256 pixels. To control for missing coverage within urban areas, as defined in Fig. 2, we filter out grid cells that have less than 10% of their pixels covered with buildings. This results in 1,035 tiles of 256 × 256 pixels or  ~2,560 × 2,560 m. The train/validation split ratio is 90%/10%. For testing, we primarily use the city of Shenzhen. Since the latter is a large city with a diverse urban fabric, it arguably serves the testing purpose well and we deem its reference coverage quality relatively high.To assess the generalizability and transferability of our model to Chinese cities overall, we extend the test set by another 15 cities beyond Shenzhen in an alternative approach. The extended test set additionally includes Chongqing, Dalian, Dongguan, Guangzhou, Hangzhou, Harbin, Hefei, Nanjing, Nanning, Qingdao, Shanghai, Shaoxing, Tianjin, Wuhan, and Zhengzhou. These test cities are not in the training-data set, to ensure the independence of the training- and test-data samples. For each of those cities we select a trustworthy image area by manual verifying against Maxar’s 0.5m satellite imagery^31^ to ensure a high accuracy with over 90% of the buildings correctly labeled in the selected zones. Only these high-quality-data areas are used for evaluation. The trustworthy reference area of Shenzhen covers 1,099km^2^ and the trustworthy reference data of the extended test set covers 9,795km^2^.

### Multi-task learning

For building-footprint prediction, we formulate the task as a binary classification problem, where each pixel is labeled as either part of a building or the background. We approach building-height estimation as a regression, where a continuous value for building height is predicted, conditional on the presence of a building at that location (footprint). We adopt a multi-task learning setup in which both objectives are learned jointly within a unified training pipeline.

The total training loss is based on a weighted sum of the footprint-classification and height-regression components.These two losses operate in different units. Classification loss compares binary labels to predicted probabilities, and regression loss compares height values normalized to the [0,1] range by dividing by 400 (the maximum building height in the dataset). Extensive hyperparameter tuning has resulted in equal weights for both loss components in our final setup. To ensure that the model learns to estimate building height only where it is relevant, the regression loss is applied selectively: height loss is only computed for pixels identified as likely buildings by the footprint-classification output. For footprint classification, Dice loss is used consistently across all models. The specific formulation of the height-regression loss varies by model and is detailed below.

Data augmentation is uniformly applied and includes random rotations (by  ±10 degrees), affine transformations (shear, scale, translation), and random deletion of pixels targeted mainly at Sentinel-1 inputs to mimic sensor noise and cloud occlusion.

We present and evaluate seven model variants (see Table 1). All are trained for 500 epochs and use a fixed training configuration: Adam optimizer with a learning rate of 0.003, weight decay of 0.0001, and a learning-rate decay at epoch 50 by a factor of 0.1. Across models, training was performed on a server with 64 AMD EPYC 7313 cores, 504 GB RAM, and a NVIDIA GeForce RTX 3090 GPU.

**Table 1:** Key Distinct Configuration Attributes per Model.

Model Name	Encoder	Decoder	Structure	Reg Loss	Cls Loss
UNet + Berhu/Dice	ResNet34	UNet	Single-stage	Berhu	Dice
UNet + Weighted L1	ResNet34	UNet	Single-stage	Height-weighted L1	Dice
Unified Two-Stage	ResNet34	UNet	Two-stage Unified	Berhu	Dice
Decoupled Two-Stage (Cls)	ResNet34	UNet	Two-stage Decoupled (Cls)	—	Dice
Decoupled Two-Stage (Reg)	ResNet34	UNet	Two-stage Decoupled (Reg)	Berhu	Pretrained
Swin-UNet Multi-Task	Swin	UNet	Single-stage	Berhu	Dice
SegFormer Multi-Task	MiT-B2	SFHead	Single-stage	Berhu	Dice
Swin Two-Stage + Weighted L1	Swin	UNet	Two-stage Unified	Height-weighted L1	Dice

The applied models differ primarily in architecture, task formulation, and loss configuration. The simplest baseline, **UNet + Berhu + Dice**, uses a standard UNet architecture^32^ with a ResNet-34^33^ encoder and two output heads trained – one for footprint classification and one for height regression – jointly using Dice loss and Berhu loss^34^. The **UNet + Weighted L1** variant uses the same architecture but replaces the Berhu loss with a height-weighted L1 loss during training, placing more weight on errors in taller structures. With the height-weighted L1 loss, we choose height-weight power and height-weight bias as the balancing parameters. They control how strongly the regression loss emphasizes tall versus short buildings: the power determines how much the weight grows with building height, while the bias ensures that also small buildings contribute to the loss. By tuning these two values, we can shift the training focus toward tall structures (higher power, lower bias) or distribute weight more evenly across all height levels.

Building on these baselines, the **Unified Two-Stage** model introduces a conditional prediction mechanism. It shares a single encoder-decoder backbone and separates footprint classification and height regression into two sequential stages. In the first stage, it produces footprint-class probabilities. In the second stage, the input image is passed again through the same encoder and decoder. The resulting decoder features are concatenated with the original input along the channel dimension and fed to a dedicated, shallow regression head. The final height predictions are then masked using the predicted building regions (i.e., only class-1 pixels are retained), enforcing that non-building areas are explicitly zeroed out during inference. While all models restrict regression loss to building regions, this model uniquely integrates the masking directly into the forward pass, ensuring spatial sparsity in the output by design and encouraging the regressor to focus solely on semantically relevant areas.

In contrast, the **Decoupled Two-Stage** model splits the footprint classification and height regression into two entirely separate models. The footprint classifier is trained independently and frozen. Its output is then used as an input for the height-regression model. This approach isolates learning across tasks but enables clearer architectural decoupling. The footprint-classification and height-regression tasks are represented in Table 1 as separate rows.

The **Swin-UNet Multi-Task** model integrates a Swin Transformer^35^ encoder with a UNet-style decoder in the standard multitask framework. It preserves the dual-head setup but benefits from the hierarchical vision-transformer architecture,allowing better capture of meso-spatial dependency patterns. The **SegFormer Multi-Task** model replaces both the convolutional and UNet-style components with a pure transformer-based encoder-decoder design. It uses a MiT-B2 backbone and SegFormer head^36^ to jointly predict footprint-class and building-height maps.

Finally **Swin Two-Stage + Weighted L1** combines several attributes of the previously described models. It utilizes the Swin encoder with the conditional prediction mechanism of the **Unified Two-Stage** model and the height-weighted L1-loss implementation for its regression loss.

## Data Records

The dataset^37^ is freely available from the Harvard Dataverse repository at 10.7910/DVN/3LTFEW. It was generated with the **Swin Two-Stage + Weighted L1** model described in the previous section. The prediction is in the original Sentinel resolution of  ~10 × 10 m, measuring the average estimated building height per pixel. We recommend aggregating the raster depending on the desired precision by referring to 48 and applying a temporal kernel (e.g., mean or median) for smoothing across years if necessary. We provide a file with annual data in GeoTIFF format with the world geodetic system 1984 coordinate reference system (EPSG:4326) for 106 cities. The set of cities we estimate building stocks for is defined as follows: the city either belongs to our training data (the 37 cities mentioned above) or it belongs to the set of first, second, third, or fourth tier cities according to the Ranking of Cities’ Business Attractiveness in China 2022^38^. We define the spatial area of each city as the bounding box of the union of the corresponding entry in the ESRI Urban Areas^39^ (with an additional buffer of 10 km) and the bounding box of our building reference data. If the spatial extent is not defined by either of these two data sets, we delete the city from the list. Figure 2 illustrates the bounding boxes of the 106 cities in our data set. City years with lower then 66% of pixels covered by sufficiently high quality Sentinel 2 imagery are excluded.

## Technical Validation

### Model evaluation

We evaluate models using pixel-level metrics for footprint segmentation and continuous error metrics for building-height estimation. For footprints, we report precision, recall, and the dice coefficient (F1), which is the harmonic mean of precision and recall. For height estimation, we evaluate the performance using the root mean squared error in meters (RMSE), Aggregated RMSE in cubic meters (Agg. RMSE) representing building-volume prediction errors at a 100m grid, and the high-rise RMSE in meters regarding pixels with buildings of over 40 m in height in the reference data. The three height metrics provide complementary perspectives: simple RMSE captures per-pixel accuracy, Agg. RMSE reflects practical usability in building-volume analysis by combining footprint accuracy and height in an aggregated manner, and high-rise RMSE focuses on the performance in predicting tall structures, which is known to be the most challenging task.

Table 2 compares a range of baseline and advanced models in the test trustworthy test set area in Shenzhen. As a baseline, only pixels with constant footprint and height predictions using the median (0m, no building) and mean (2.4m, only buildings) heights are included. The mean predictor trivially achieves perfect recall (1.0) for footprints but exhibits poor precision and a relatively high RMSE, confirming that it indiscriminately assigns buildings to all pixels. The **Random Forest** model^14^, trained on the same Sentinel-1 and -2 inputs, serves a similar purpose but performs notably worse than deep learning models. It achieves high recall (0.776) but low precision (0.128), indicating excessive overestimation of footprints. Its height predictions are also the least accurate across the board.

**Table 2:** Results of all models on the Trustworthy Test Set (Shenzhen City).

Method	Precision	Recall	Dice (F1)	RMSE (m)	Agg. RMSE (m^3^)	High-Rise RMSE (m)
Median (0 m)		0		10.06	50575	79.4
Mean (2.4 m)	0.128	1	0.226	9.73	43449	77
Random Forest	0.364	0.776	0.496	11.01	52629	59.7
UNet + Berhu/Dice	0.5	0.569	0.533	8.85	29265	57.8
UNet + Weighted L1	0.482	0.609	0.538	8.96	29065	56
Unified Two-Stage	0.495	0.604	0.544	9.18	29223	54.5
Decoupled Two-Stage	0.47	0.571	0.516	9.49	33040	53.6
Swin-UNet Multi-Task	0.477	0.668	0.557	8.97	29178	55.8
SegFormer Multi-Task	0.446	0.622	0.52	8.77	29379	57.8
Swin Two-Stage + Weighted L1	0.474	0.653	0.549	8.81	27664	56

Top-performing values are bolded, second-best are underlined, and third-best italicized. Precision, Recall, Dice, RMSE, and High-Rise RMSE are measured at the pixel level, with RMSE and High-Rise RMSE expressed in meters; High-Rise RMSE is computed only for pixels where the reference building height exceeds 40 m, while Aggregated RMSE is reported in cubic meters per 100 m cell representing building volume.

The **UNet + Berhu + Dice**^34^ performs solidly. The **UNet + Weighted L1** shows a competitive Dice-score value (0.538) and achieves the second-best Agg. RMSE value (29,065 m^3^), indicating its superior performance in volumetric accuracy. This confirms that L1-style losses better capture the distribution of height values, especially for lower- to mid-rise structures.

Two-stage models, regarding both unified and decoupled variants, show stronger performance for high-rise buildings: the best high-rise RMSE of 53.6 m is obtained with the Decoupled Two-Stage model. This aligns with the architectural design, where the second-stage regression operates only on positive-footprint-classified pixels, reducing noise and improving accuracy for the prediction of taller buildings.

Transformer-based architectures show promise. The **Swin Multi-Task** model achieves the highest Dice score for footprint (0.557) and the third-best Agg. RMSE for building volume (29, 178 m^3^). This highlights Swin’s strength in spatial footprint delineation, which supports better aggregated volume predictions. On the other hand, the **SegFormer Multi-Task** model achieves the best overall RMSE (8.77 m), but it struggles notably with high-rise detection.

**Swin Two-Stage + Weighted L1** successfully unifies several of the strengths of the other model architectures. With the second-best Dice score (0.549) and the second-best RMSE (8.81 m) on the pixel level. Regarding building volume it strongly outperforms all the other models with the best Agg. RMSE of 27,664 m^3^.

Overall, the performance spread across models is fairly narrow. All models perform similarly well in both footprint detection and height estimation.

**Fig. 3:**
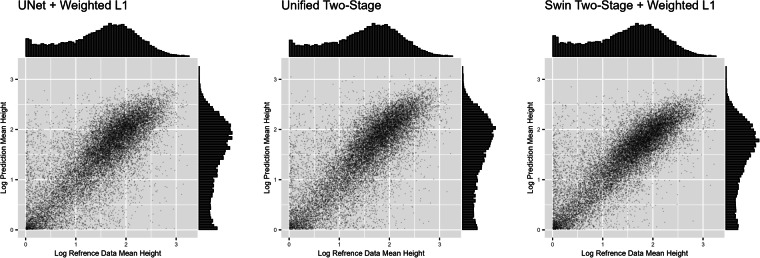
Comparing Scatter Plots of Logarithmic Mean Height Plus One when Aggregated to  ~200 × 200 m cells. The trustworthy reference data are shown on the x-axis and predicted height on the y-axis. Only cells with non-zero values for both dimensions are shown.

These findings are confirmed by Fig. 3. While purely UNet-based architectures outperform the Swin-based ones in the most dense areas (i.e., ones with high-rise buildings) the frequently occurring areas with mid-range density are better captured by the latter.

**Fig. 4:**
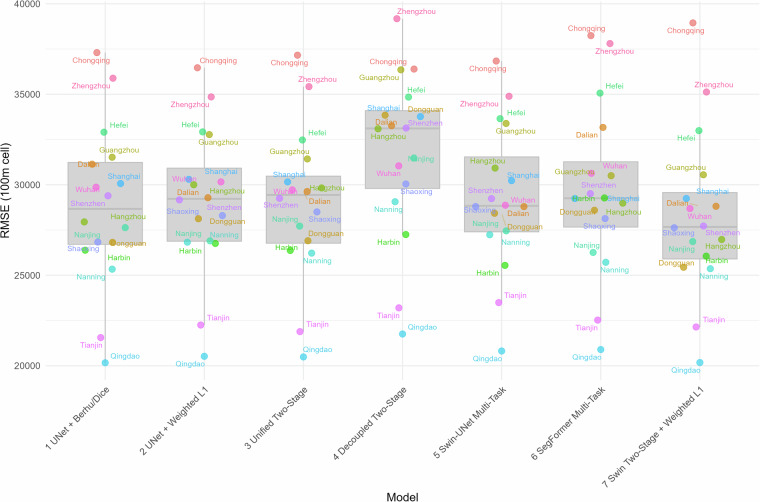
Root Mean Square Error (RMSE) aggregated over 100-cell units (in cubic meters) for each model across multiple test cities. Each point represents the aggregated RMSE for a single city, with quasi-random jitter added for visual clarity. The boxplots summarize the distribution of RMSE values per model, providing a comparative view of model generalizability. Lower RMSE values indicate better performance in predicting spatial quantities across diverse urban contexts.

Figure 4 presents the distribution of Agg. RMSE values (per 100m cell) across multiple models and test cities. The boxplots reflect the general range and variability of model performance, while individual cities are overlaid as jittered points and labeled to reveal city specific patterns. Shenzhen appears consistently close to the median RMSE across models. Due to the large area and the rich pattern of urban fabrics it covers, it is broadly representative of the overall test distribution and, hence, provides an appropriate benchmark for performance evaluation. Across most models, the differences in Agg. RMSE for building volume are relatively minor, with no statistically significant outliers – except for the **Decoupled Two-Stage** model, which consistently performs worse. The observed Agg. RMSE values range approximately between 20, 000m^3^ and 40, 000m^3^, indicating moderate but acceptable generalization performance to unseen urban areas. Notably, the variance in Agg. RMSE between cities is not strongly correlated with their median error levels, which implies that the differences may stem more from variation in local data quality than from inherent weaknesses in the models themselves.

Given the objectives of this study – to recover urban volume and footprint distributions over large areas and over time – we focus on Agg. RMSE as the most relevant performance measure. We select the **Swin Two-Stage + Weighted L1** model with height-weight-power parameter 0.5 and height-weight-bias parameter 1 as our preferred model. We also tune the weighting parameter of classification and regression losses, where an equal weighting of both as 1 has the best performance. It offers a balanced trade-off between footprint Dice-loss accuracy and volumetric consistency, while remaining computationally efficient and robust across the evaluated urban landscape. During prediction we adopt a sliding-window approach where only the central part of each image (128 × 128 pixels) is predicted only once, as suggested in Figure 4 of^34^.

### Limitations

**Fig. 5:**
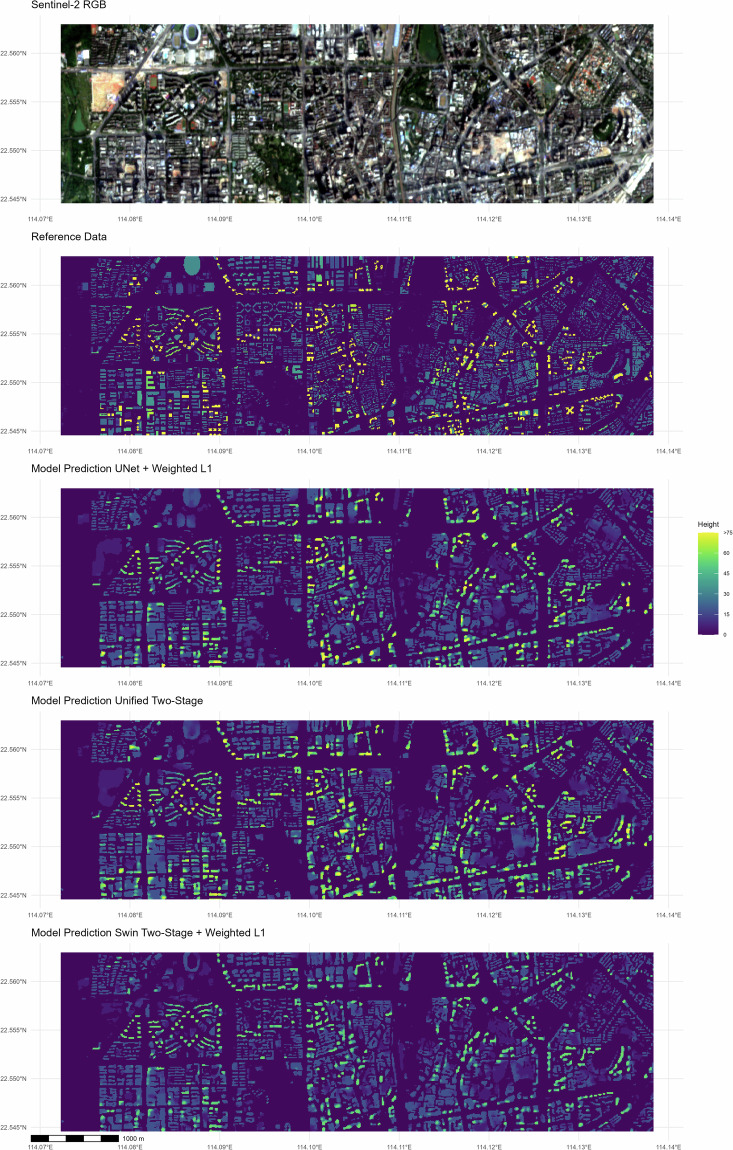
Visualization of Three Models’ Prediction in Shenzhen along with the corresponding Reference Data and the Sentinel 2 RGB bands.

**Fig. 6:**
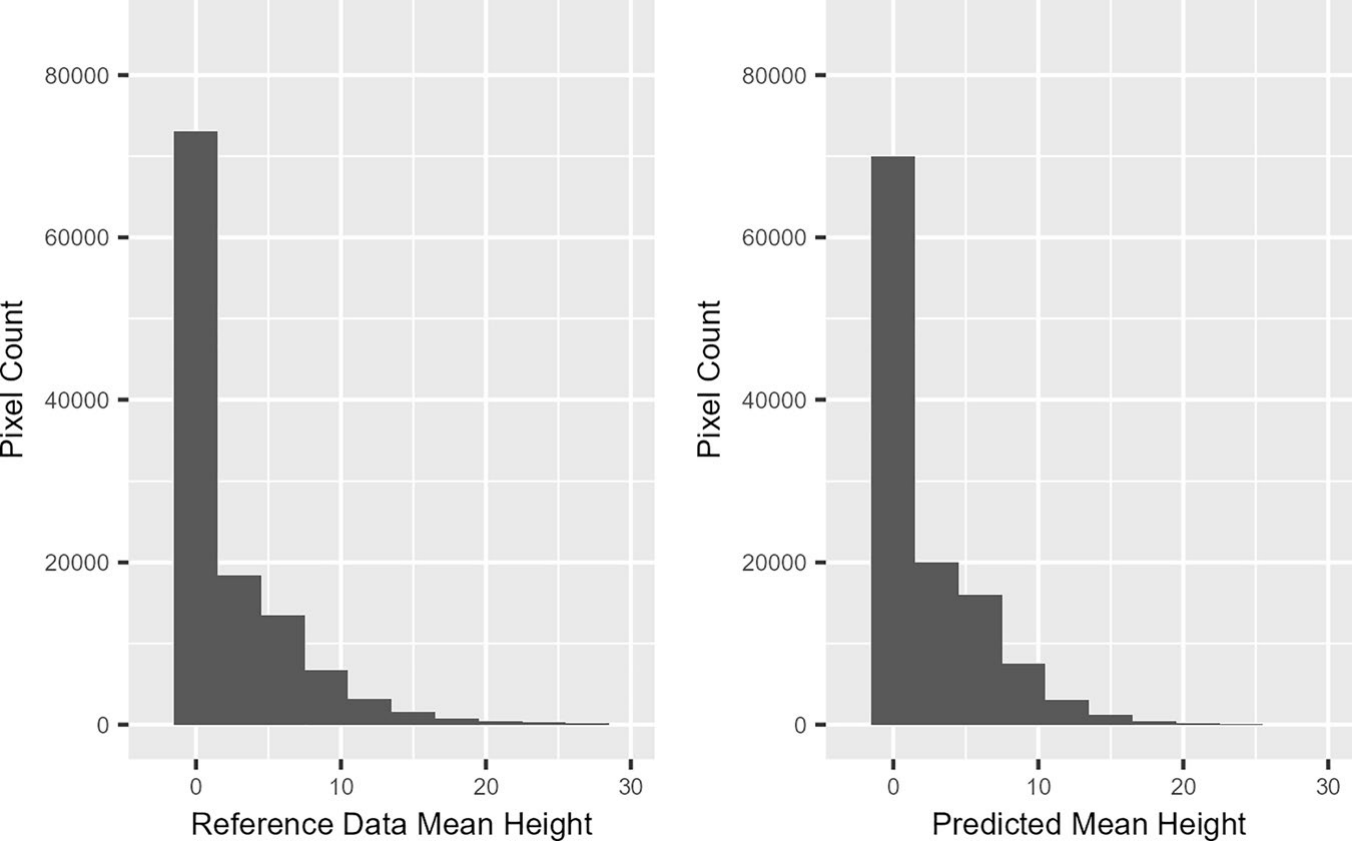
Histogram of the Pixel-wise Height Distribution for Shenzhen in the Trustworthy Reference Data vs. the Prediction Up to 100 m.

From visual inspection (see Figs. 5 and 6) we identify two primary challenges. First, in terms of footprint, all of our sampled tiles successfully identify the regions covered with buildings. However, our model encounters difficulties in accurately distinguishing individual buildings that are small and located close to each other, leading to a slight upward bias of the building-footprint coverage. This is largely due to the inherent limitation of Sentinel’s resolution in relation to the urban topology. In our designated reference area in Shenzhen, approximately 91% of the buildings are located within a distance of less than 10m from their nearest-neighboring building, corresponding to the size of a single pixel. Furthermore, 54.6% of the buildings are within 10m of at least two other buildings. However, the inter-building area will be of lesser relative importance when aggregating to a somewhat coarser grid. Therefore, we propose that evaluating the prediction results at a more aggregated level than the pixel would yield more meaningful insights for most relevant applications.

Second, from the perspective of height estimation our predictions accurately portray the hierarchical structure of building heights. However, they consistently underestimate the height of high-rise buildings.

### Comparison with other predictions

**Fig. 7:**
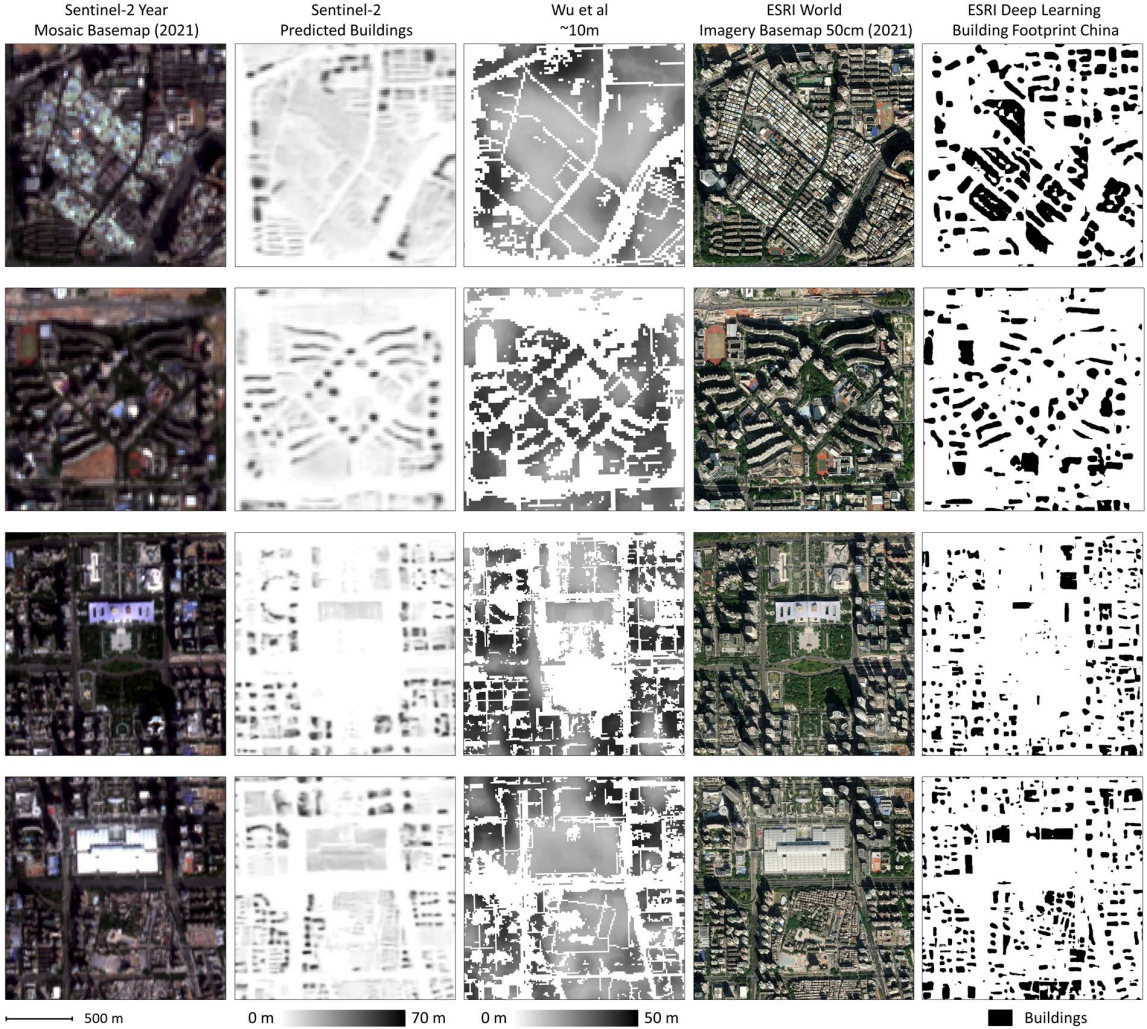
Comparison among Our Predictions, Wu *et al*.^14^, and ESRI Building Footprint Model in China.

Figure 7 displays four scenes of typical urban fabric in Shenzhen. We compare the Sentinel-2 real color bands in the leftmost column, then our prediction in the second column, the prediction for footprint and height with the RF-model by Wu *et al*. in 2023^14^ in the third column, a snapshot from the ESRI World Imagery Basemap in the fourth column, and a prediction of the ESRI’s pre-trained model on Building Footprint Extraction - China^40^ in the last column. The latter takes the high-resolution imagery in the fourth column in its default setting as an input. The overall structure of the buildings is generally well captured by all the models. While the RF tends to blur all buildings into monolithic street blocks, the ESRI footprints are clearly defined. Our footprint constitutes a middle way. There, actual footprints are wide, similar to RF, but at their edges they fade out with a very low building height predicted. In terms of height prediction, our model discriminates buildings, while the RFmodel produces a more smoothed-out topology, blurring building boundaries.

We can also observe that both models that use a  ~10 × 10 m resolution, ours and RF, struggle to delineate small adjacent buildings, as mentioned above, as those buildings are sometimes smaller than the image resolution. While our model predicts the height and orientation of each building comparatively well, both the RF model and the ESRI model miss much of the overall structure. Our model strongly outperforms the ESRI model in differentiating squares and roads from buildings, according to the tile examples shown in the third and fourth rows. Surprisingly, the ESRI pre-trained model clearly misses large footprints in white color and has trouble telling apart buildings from greenery when they are adjacent.

### Evaluation in aggregated grids

**Fig. 8:**
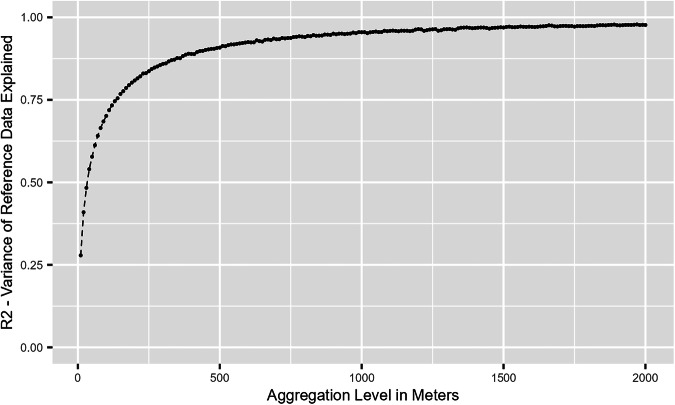
Share of Explained Variance of Building Height in the Reference Data by Model-predicted Building Height as a Function of the Aggregation Level. X-Axis: Side length of grid cell in meters. Y-Axis: Proportion of trustworthy reference data building height variability explained by a correlation model informed by the model prediction.

Here, we evaluate the predictions on more aggregated grids. Table 2 illustrates that the pixel-level precision is modest on the  ~10 × 10 m grid with numerous false positives among the identified building footprints. However, it matters where the false positives occur spatially. It turns out that this is the case mostly in the immediate geographical neighborhood of correctly identified buildings, where the buildings fade out toward the grid edges and, the incorrectly identified footprints tend to have a very low height. Therefore, we illustrate the model performance with higher spatial aggregation to quantify this effect. Figure 8 shows the R-squared of a simple correlation between our predicted building heights and the reference data depending on spatial aggregation (measured in terms of the side length of the spatial grid cells) for Shenzhen in 2019. The x-axis represents grid-cell side lengths in 10 m intervals (each dot in the figure corresponds to one such interval). The evidence attests to a sharp improvement of the predictive power of the model as spatial resolution declines starting from a  ~10 × 10 m grid. On that grid, our model explains about 31% of the variation in true building heights in the reference data. At the  ~200 × 200 m grid, the explanatory power increases to over 80%. The R-squared converges to around 98% with a  ~2,000 × 2,000 m aggregation and beyond. This reflects how well the proposed model predicts the actual heights of the buildings on the spatial meso scale. Given the image quality of Sentinel, one often focuses on meso-aggregated levels (such as ones on a  ~200 × 200 m grid) of the floorspace density, as the latter offers sufficiently-precise indicators of urban development. While we provide the original  ~10 × 10 m estimation as a public data set, depending on the precision needed in a particular application, we suggest aggregating the data raster according to Fig. 8.

### Correlations with night-light data

Over the past two decades, night-light (NTL) satellite (remote-sensing) imagery became a workhorse measure in urban and regional studies, demography, and economics. NTL data have been used extensively to measure city structure^41,42^, population density^43,44^, economic activity and growth^45–49^, and poverty and wealth^50,51^. These data are (i) available throughout the world for a relatively long time span of several decades; and (ii) they are measured with comparable or identical precision throughout space and time. On the contrary, more exact measures of demography, economic activity, or urban and regional development are often measured at much lower frequency (e.g., census data at the decade level), in a selected way (samples are drawn with reporting thresholds; or only selected jurisdictions report specific data), and the reporting is not conducted with the same quality or at the same time across jurisdictions. A disadvantage of NTL data relative to the ones generated in this paper is that they do not come at a similarly fine-grained spatial resolution as the Sentinel and other daylight satellite imagery. NTL data are often at a  ~1,000 × 1,000 m resolution^45^, and only more recent NTL data are available on a  ~700 × 700 m resolution^48^.

**Fig. 9:**
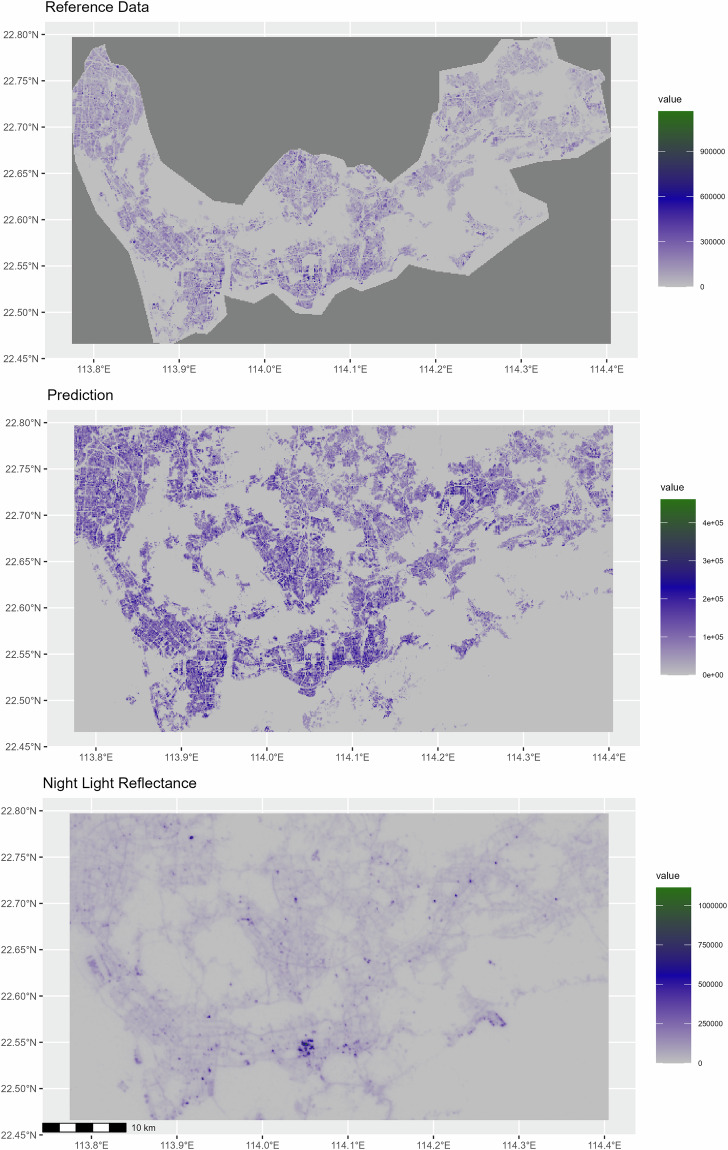
All Reference Data, Model Predictions, and Luojia NTL Data for Shenzhen 2019.

Their widespread use and availability made NTL data a natural candidate to compare our predictions with. Figure 9 shows the comparison of three data samples of Shenzhen, all the reference data for Shenzhen (masked with he hand-drawn trusted areas that define the test set), our prediction by the best performer and Luojia 1-01 NTL data^52^ radiance intensity. The reference data and our predictions are shown with average heights per cell aggregated to a uniform 120 × 120 m scale for consistency with the NTL data. The illustration clearly indicates that the three data sets describe a similar urban structure. A robust correlation exists between NTL radiance and the predicted micro-regional building volume.

**Fig. 10:**
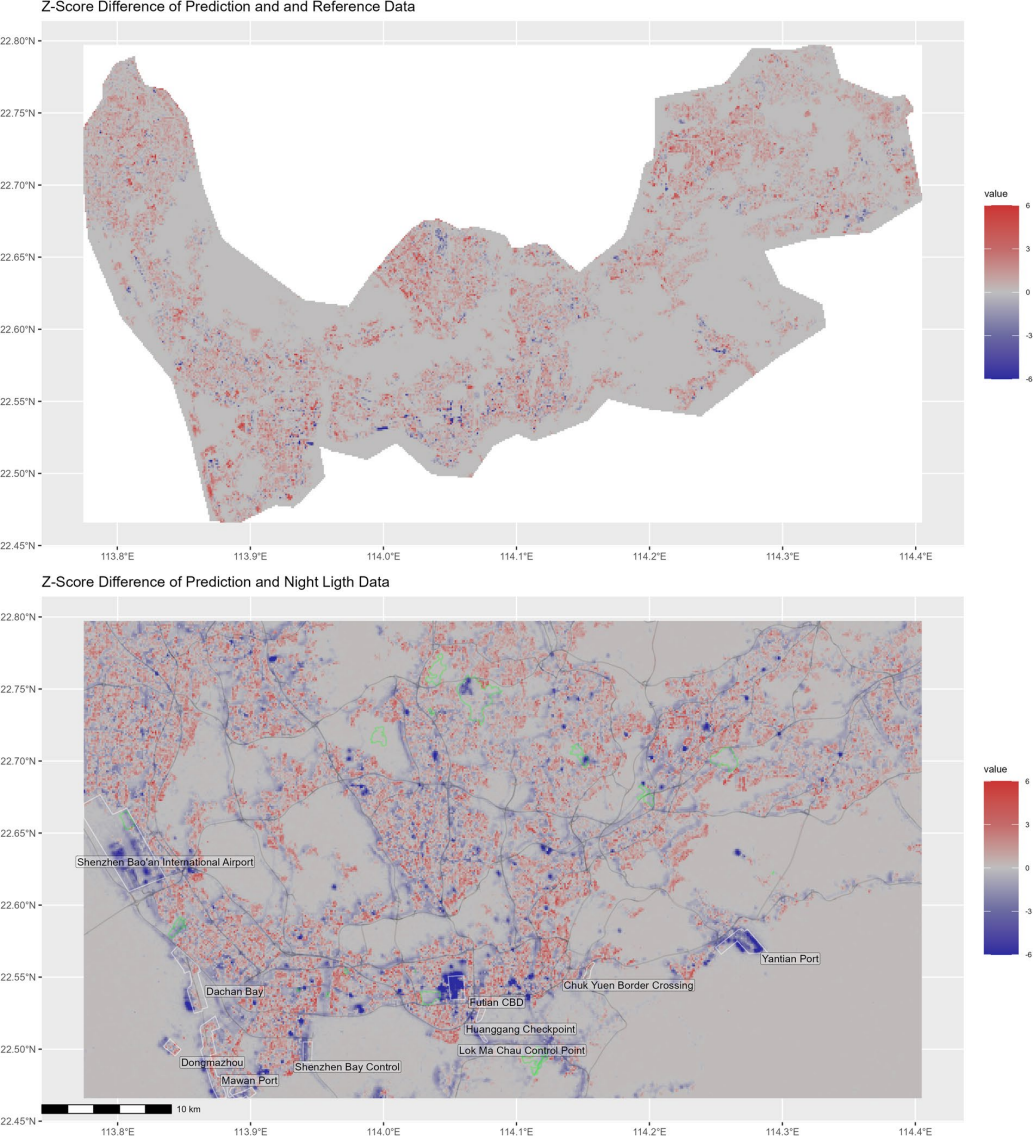
Difference Map between Model Predictions and Reference Data (Upper Map) as well as Model Predictions and Nightlight Data (Lower Map). All values are *z*-standardized before the differences are calculated. Red zones indicate an over-estimation of our model compared to the reference data and an under-estimation of the NTL data relative to our model. Purple zones indicate an under-estimation of our model relative to the reference data and an over-estimation of the NTL data relative to our model. Black lines: highways from OpenStreetMap (OSM), green contours: golf courts, white contours: selection of infrastructure with nightlight radiance.

Figure 10 shows the difference between our prediction and the trustworthy reference data in Shenzhen (upper map) and the difference between our prediction and the NTL data (lower map). To make the data comparable, they are *z*-score-standardized before calculating the difference. The lower map also shows highways (in black) as found by OpenStreetMap (OSM), a selection of infrastructure features (in white), and golf courses (in green). The red zones indicate an over-estimation of our prediction compared to the reference data (upper map) and a relative under-estimation of the NTL data (lower map), while the purple zones indicate an under-estimation of ours relative to the reference data (upper map) and a relative over-estimation of the NTL data (lower map). Visual inspection shows that the red regions on the upper map mostly indicate missing buildings in the reference data. Purple regions, on the other hand, are caused by two factors, either inaccuracies of the reference data or height under-estimation bias on the part of our model. The lower map reveals some shortcomings of the NTL data. Most importantly, they heavily over-represent large-scale infrastructures such as ports, customs checks, airports, highways, as well as recreational facilities such as golf courts that are illuminated during the night. On the contrary, NTL data underestimate building volumes in central parts of the city with more high-rise buildings as well as in other very dense urban environments.

The deviation of building volumes from the NTL intensity underlines the need of a robust pipeline of the proposed sort to improve research that so far has relied mainly on NTL data. We see from Fig. 10 that our measure and NTL data highlight different aspects of the urban environment. The former gives a better overview of building and floorspace density, while the latter emphasises large-scale infrastructures and roads. NTL may further deviate from population or economic density along cultural or regulatory factors and generally underestimate dense urban areas compared to medium-dense sub-urban areas with a higher share of road surface coverage^53^. In particular, good approximations of population density and building measures that require a more fine-grained spatial resolution cannot be obtained without bias from the NTL data. In conclusion, NTL data capture residence-based and buildings- as well as floorspace-related measures of urban density with a considerable bias towards infrastructure. Moreover NTL data underestimate the presence and relevance of non-manufacturing, non-transport-related economic activity that does not intensively use infrastructure.

## Usage Notes

We deliver a data set with building-footprint and -height prediction at the original Sentinel resolution of  ~10 × 10 m. We provide a file in GeoTIFF format for each year with the coordinate reference system “EPSG:4326” for 106 cities. To use the data at large, we recommend that users choose a reasonable cut-off for aggregation based on Fig. 8, depending on the precision requirement in meso-level regional studies.

## Data Availability

The Building Volume Panel of 106 Chinese Cities 2018-2023 Dataset Version 4^37^ described in this study is publicly available from the Harvard Dataverse repository at 10.7910/DVN/3LTFEW. The dataset is provided in GeoTIFF format, with one file for each of the 106 cities. Yearly predictions are provided as layers within each file. Additional details on the dataset contents, versions, and variables are described in the Data Records section.

## Code availability

Our codebase is publicly available at https://gitlab.ethz.ch/spapini/sentinelheightnet. The code is written in Python 3.8 and is structured as follows:

1. **Model Training**: We provide training scripts that allow users to reproduce all models presented in this work by modifying the configuration file.

2. **Best Performing Model**: We release the weights of our best-performing model. Given Sentinel-1 and Sentinel-2 imagery, it can be used to predict building heights in any region. (Note: we advise against applying the model outside of Chinese cities, as it was trained and tested specifically on those.)

3. **Model Inference**: We include a complete prediction pipeline, featuring a sliding window mechanism for efficient large-scale inference.
